# Phantom Limb Pain: Low Frequency Repetitive Transcranial Magnetic Stimulation in Unaffected Hemisphere

**DOI:** 10.1155/2011/130751

**Published:** 2011-05-11

**Authors:** Andrea Di Rollo, Stefano Pallanti

**Affiliations:** ^1^Department of Psychiatry, University of Florence, 50134 Florence, Italy; ^2^Institute of Neurosciences, 50137 Florence, Italy; ^3^Department of Psychiatry, Mount Sinai School of Medicine, New York, NY 10029, USA

## Abstract

Phantom limb pain is very common after limb amputation and is often difficult to treat. The motor cortex stimulation is a valid treatment for deafferentation pain that does not respond to conventional pain treatment, with relief for 50% to 70% of patients. This treatment is invasive as it uses implanted epidural electrodes. Cortical stimulation can be performed noninvasively by repetitive transcranial magnetic stimulation (rTMS). The stimulation of the hemisphere that isn't involved in phantom limb (unaffected hemisphere), remains unexplored. We report a case of phantom limb pain treated with 1 Hz rTMS stimulation over motor cortex in unaffected hemisphere. This stimulation produces a relevant clinical improvement of phantom limb pain; however, further studies are necessary to determine the efficacy of the method and the stimulation parameters.

## 1. Introduction

Phantom limb pain (PLP) is very common after limb amputation and has a reported incidence of up to 87% of amputees [1]. This type of pain can be difficult to treat and usually responds poorly to conventional pain treatments [2–4]. Conversely, the electrical stimulation of the primary motor cortex (M1) has proved to be an effective treatment for intractable deafferentation pain. This treatment started in 1990, and many patients have been treated up to now. The patients who have been operated on were suffering from poststroke pain (59%), trigeminal neuropathic pain, brachial plexus injury, spinal cord injury, peripheral nerve injury, and phantom limb pain [5]. This treatment consists in chronic motor cortex stimulation (MCS) through implanted epidural electrodes. It results invasive and outcome varies from patient to patient [6, 7]. Otherwise cortical stimulation can be performed non-invasively by transcranial magnetic stimulation (TMS). A number of studies have shown that a single session of repetitive transcranial magnetic stimulation (rTMS) can relieve pain transiently in some patients with chronic neuropathic pain [8–10]. Other studies have shown that the duration of pain relief can be extended by repeated application of rTMS every day for five days in patient with trigeminal neuralgia and poststroke pain syndrome [6] or for ten days in patients with fibromyalgia [11]. In contrast, one study failed to see any long-term therapeutic effect of three weeks daily parietal cortex rTMS in two patients with phantom limb pain [12]. The majority of studies apply high frequencies (>1) with pulses below motor threshold on motor cortical area corresponding to the hand of the painful side. The reason for this is twofold: epidural stimulation usually employs pulses below motor threshold at ~40 Hz. and a study shows that the applications of rTMS at high frequency is more effective than applications of rTMS at low frequency (≤1) in this area of stimulation [8]. However, the effect of stimulation in unaffected hemisphere for phantom limb pain remains unexplored. In other cases, like neglect or recovery in stroke, stimulation with low frequency rTMS in unaffected hemisphere have shown therapeutic properties [13, 14]. We report a case of phantom pain limb treated with 1 Hz stimulation over motor cortex in unaffected hemisphere.

## 2. Case Study

**Figure 1:**
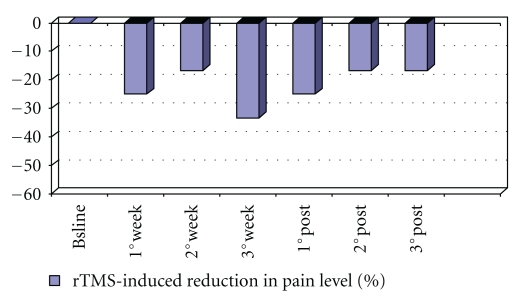
The graph shows the reduction in percentage of pain in time. The percentage of pain level modification was calculated from the VAS score by the following equation (post.rTMS − pre.rTMS pain scores) × 100/(pre.rTMS pain scores).

A 36-yr-old, right-handed man, who had had a motorbike accident ten years ago, had total surgical amputation of the left arm. At the time of his arrival to our institute, he had perception of phantom limb and was experiencing severe phantom limb pain. This perception and pain have existed immediately after the amputation. The perception of phantom limb was always in the same position near the chest with the hand partially closed near the shoulder. The patient experienced pain like paresthesia, dysaesthesia and burning sensation especially in phantom thumb, index and medium, and in the total phantom limb too. The pain was present every day, always in wakefulness but not in sleep. Such pain persisted during the day and sometime became very intense for some seconds. In the past, the patient tried antiepileptic drugs, tricyclic and SSRI antidepressant, anti-inflammatory-analgesics, and opioids in order to relieve the pain. At the time of his arrival to our institute, he was having the best therapy that gave a partial relief to the pain. The therapy consisted in methadone 30 mg/day and pregabalin 300 mg/day. Neurological examination showed miosis and light ptosis in left eye, like Bernard-Horner syndrome which has existed immediately after the amputation. The examination also showed tactile hypoesthesia in the surgical scar, while the tactile stimulation of the area near the scar increased the pain in the phantom limb. The tactile stimulation of the left side of the face increased the pain in the phantom limb too. Chest MRI and CT with contrast excluded a peripheral component of pain due to a concomitant lesion of the inferior brachial plexus. The patient gave the written informed consent. At the baseline, at the end of every week, for three weeks during treatment and at the end of every week for three weeks after treatment, the following tests were administered: Hamilton Rating Scale for Depression (HAM-D), Hamilton Rating scale for Anxiety (HAM-A), Mania Rating Scale (MRS), CORSI TEST, Phonemic Verbal Fluency, and Visual Analogue Scale (VAS) for pain-0 (no pain) and 10 (maximal pain). The percentage of pain level modification was calculated from the VAS score by the following equation (post.rTMS – pre.rTMS pain scores) × 100/(pre.rTMS pain scores).  Figure 1 below shows the reduction in percentage of pain in time. Clinical Global Impression-Improvement scale (CGI-I) was evaluated at the end of the third week of treatment. rTMS sessions were conducted in a laboratory staffed by physicians certified in basic life support and trained in the prompt recognition and treatment of seizures and other medical emergencies. Repetitive TMS was administered using a MAGSTIM rapid magnetic stimulator (Magstim Company, Ltd., Whitland, U.K.). We used a 70-mm figure eight-shaped coil. Patient sat in a reclining chair with a headrest for stabilization of the head and wore protective earplugs. Resting motor threshold (RMT) was defined as the intensity required eliciting at least five MEPs of 50 *μ*V in peak-to-peak amplitude with 10 consecutive stimulations, when the coil was placed over the optimal position to activate the abductor pollicis brevis muscle in right hand based on electromyographic recording [15]. During treatment, the following were applied for 15 minutes, thirty 20-second trains at 1 Hz at 80% of RMT with a 10 seconds intertrain interval (a total of 600 stimuli per session were applied over the left motor cortex), these parameters are now widely considered safe [16]. A full course comprised fifteen daily sessions administered on weekdays, beginning on Monday. At all times, the coil was held tangentially to the scalp, with the handle pointing back and away from the midline at 45°. During every session of stimulation the patient had the sensation that the phantom limb went away from the shoulder towards mid-line in the direction of the pelvis, and the intensity of phantom limb pain reduced. The patient experienced no adverse event during or after rTMS application. At the end of the third week of treatment, the pain was reduced about 33.3% (see Figure 1), in fact VAS changed from 6 (pretreatment) to 4 (posttreatment), with CGI-I = 2 (much improved). In three weeks after treatment the percentage reduction of pain was reduced to 25% in the first week after the end of treatment and remained stable at about 16.6% in the second and in the third week after the end of treatment (Figure 1). During the three weeks of treatment and during the three weeks after treatment, Ham-D, Ham-A, and MRS all remained stable at ≤6. Also, score of the CORSI TEST remained stable at 5 and the score of the Phonemic Verbal Fluency remained stable at 17.6 and so these tests did not show cognitive impairment or improvement. RMT of unaffected hemisphere increased during treatment, in fact at baseline its value was 84% of Maximum Output of the Stimulator, after the first week of treatment, its value was 86% of Maximum Output of the Stimulator, and at the end of the second and third week of treatment its value was stable at 88% of Maximum Output of the Stimulator.

## 3. Discussion

Although the study has a strong limitation due to the absence of placebo (sham) control, it nevertheless shows that the method of stimulation in nonphantom limb hemisphere with 1 Hz stimulation ameliorates the phantom limb pain with longlasting antalgic effects. The effects of rTMS on pain are similar to effects obtained by Passard et al. [11]. Passard in his work applied high frequency rTMS in the left motor cortex of patients with fibromyalgia for two weeks. He obtained the maximum result at the end of treatment, and this result lightly decreased in followup. Also, we obtained the maximum reduction of pain at the end of treatment but in weeks after the end of treatment the relief in pain reduced. In order to improve the results it would be probably necessary to have a longer period of stimulation or other parameters of stimulation like a higher intensity of stimulation, respecting the safety guideline [16] and considering that stimulation is applied in the motor cortex area with high epileptic risk. 

The low frequency rTMS has showed antidepressant effects [17, 18], but in this case the relief in pain does not depend on mood change. In fact the mood of the patient remained stable, like the tests, Ham-D, Ham-A, and MRS show, remaining stable at ≤6. 

Instead, the low frequency rTMS is known to reduce the excitability of the stimulated motor cortex. This can increase the excitability of the controlateral motor cortex via transcallosal pathways, and so it can have analgesic effects in a way similar to the epidural motor cortex stimulation and to the high frequency rTMS of motor cortex. In fact chronic motor cortex stimulation using implanted electrodes is an effective treatment of drug-resistant pain [19], but its mechanism of action remains poorly understood. Some hypotheses resulted from electrophysiological and PET studies [20, 21]. In these studies, cerebral blood flow was found to increase in thalamus ipsilateral to the stimulated motor cortex, in the orbitofrontal and anterior cingulated gyri, the anterior insula and upper brainstem near the periacqueductal gray matter. Cingulate/orbitofrontal activation should participate in a modulation of affective/emotional component of pain, while descending activation of the brainstem should inhibit the transmission of discriminative noxious information [20, 21]. Besides, there are lines of evidence that chronic motor cortex stimulation using implanted electrodes might involve endogenous opioids system in the analgesic action. This hypothesis is supported by the demonstration that motor cortex stimulation via epidurally implanted electrodes induces changes in endogenous opioids systems in patients with neuropathic pain [22]. Furthermore, it has recently been shown that naloxone reverses the antinociceptive effects of epidural motor cortex stimulation in the rat [23]. Besides, a recent study shows the involvement of endogenous opioid systems in rTMS-induced analgesia [24]. In fact, naloxone injection significantly decreased the analgesic effects of rTMS of motor cortex stimulation, but did not change the effects of rTMS of the dorsolateral prefrontal cortex or sham. The differential effects of naloxone on motor cortex and dorsolateral prefrontal cortex stimulation suggest that the analgesic effects induced by the stimulation of these two cortical sites are mediated by differential mechanisms [24].

The physiopathology of the phantom limb pain is still an open field between various hypotheses. The two major research streams on the painful phantom limb are focused on the pivotal influence of the periphery and of the spinal cord, while the other is focused on the fundamental role of suprasegmental structures and of the cortex. These two research streams seem to be more complementary than in opposition [25]. However, the results of our paper show that phantom limb pain could be generated by altered interhemispheric balance. This theory and the consequent strategy have shown effects in stroke recovery [14] and in rehabilitation of visual spatial neglect [13]. This hypothesis is consistent with the results of Röricht et al. [26], which show higher excitability of the motor cortex contralateral to the intact arm in some patients with upper arm amputation, and higher excitability of the motor cortex controlateral to the amputated limb in other patients. Röricht says that variability in excitability in two hemispheres could depend on the site of amputation and on the time since amputation. The hypothesis of interhemisferic balance is in contrast with Schwenkreis et al. [27] and colleagues that found a significant reduction of intracortical inhibition in forearm amputees and an enhancement of intracortical facilitation in upper arm amputees on the affected side, revealing a hyperexcitability of phantom limb hemisphere. Others studies, with EEG or with single-pulse and paired-pulse TMS investigations, are necessary to evaluate excitability of the nonphantom limb hemisphere and of phantom limb hemisphere and its modification with treatment, to understand the role of excitability in phantom limb pain.

## 4. Conclusion

 1 Hz stimulation over motor cortex in unaffected hemisphere ameliorates phantom limb pain with longlasting antalgic effects. New experiments with this approach are necessary to confirm the therapeutic results and to improve them with better parameters of stimulation.
